# Supplementary material for: Nuclear Receptor Binding Protein 2 Is Downregulated in Medulloblastoma, and Reduces Tumor Cell Survival upon Overexpression
Source: Cancers (Basel). 2020 Jun 6;12(6):1483. doi: 10.3390/cancers12061483 (PMC7352854; doi:10.3390/cancers12061483)
Supplement: Supplementary file 1 [file cancers-12-01483-s001.pdf]

Supplementary Materials

Table S1. Evaluation of the fraction of stained cells and staining intensity in the brain tumor tissue array stained with an antibody to NRBP2.

#	Cell type	Intensity	Quantity	Cell type	Intensity	Quantity	Gender	Age	Diagnosis
1	Tumor cells - cytoplasmic	1	1	Tumor cells - nuclear	1	1	F	37	Anaplastic astrocytoma
2	Tumor cells - cytoplasmic	1	1	Tumor cells - nuclear	1	1	F	67	Anaplastic astrocytoma
3	Tumor cells - cytoplasmic	1	1	Tumor cells - nuclear	1	1	F	31	Anaplastic astrocytoma
4	Tumor cells - cytoplasmic	2	2	Tumor cells - nuclear	1	1	F	29	Anaplastic astrocytoma
5	Tumor cells - cytoplasmic	2	2	Tumor cells - nuclear	1	1	F	46	Anaplastic astrocytoma
6	Tumor cells - cytoplasmic	2	2	Tumor cells - nuclear	1	1	F	37	Anaplastic astrocytoma
7	Tumor cells - cytoplasmic	3	3	Tumor cells - nuclear	1	1	M	41	Anaplastic astrocytoma
8	Tumor cells - cytoplasmic	1	1	Tumor cells - nuclear	1	1	F	62	Anaplastic ependymoma
9	Tumor cells - cytoplasmic	2	1	Tumor cells - nuclear	1	1	M	32	Anaplastic ependymoma
10	Tumor cells - cytoplasmic	1	1	Tumor cells - nuclear	1	1	M	80	Anaplastic meningioma
11	Tumor cells - cytoplasmic	1	1	Tumor cells - nuclear	1	1	M	83	Anaplastic meningioma
99	Tumor cells - cytoplasmic	2	1	Tumor cells - nuclear	2	1	M	53	Anaplastic meningioma
12	Tumor cells - cytoplasmic	1	1	Tumor cells - nuclear	1	1	F	24	Anaplastic oligoastrocytoma
13	Tumor cells - cytoplasmic	2	4	Tumor cells - nuclear	1	1	M	34	Anaplastic oligoastrocytoma
14	Tumor cells - cytoplasmic	2	6	Tumor cells - nuclear	1	1	M	69	Anaplastic oligoastrocytoma
15	Tumor cells - cytoplasmic	3	1	Tumor cells - nuclear	1	1	F	61	Anaplastic oligoastrocytoma
16	Tumor cells - cytoplasmic	3	1	Tumor cells - nuclear	1	1	M	34	Anaplastic oligoastrocytoma
100	Tumor cells - cytoplasmic	2	1	Tumor cells - nuclear	2	1	M	49	Anaplastic oligoastrocytoma
17	Tumor cells - cytoplasmic	1	1	Tumor cells - nuclear	1	1	M	40	Anaplastic oligodendroglioma
18	Tumor cells - cytoplasmic	1	1	Tumor cells - nuclear	1	1	F	70	Anaplastic oligodendroglioma
19	Tumor cells - cytoplasmic	1	1	Tumor cells - nuclear	1	1	F	42	Anaplastic oligodendroglioma
20	Tumor cells - cytoplasmic	1	1	Tumor cells - nuclear	1	1	F	34	Anaplastic oligodendroglioma
21	Tumor cells - cytoplasmic	2	1	Tumor cells - nuclear	1	1	F	60	Anaplastic oligodendroglioma
22	Tumor cells - cytoplasmic	2	2	Tumor cells - nuclear	1	1	M	42	Anaplastic oligodendroglioma
23	Tumor cells - cytoplasmic	2	4	Tumor cells - nuclear	1	1	F	31	Anaplastic oligodendroglioma
24	Tumor cells - cytoplasmic	1	1	Tumor cells - nuclear	1	1	F	57	Atypical meningioma
25	Tumor cells - cytoplasmic	1	1	Tumor cells - nuclear	1	1	F	82	Atypical meningioma
26	Tumor cells - cytoplasmic	1	1	Tumor cells - nuclear	1	1	F	57	Atypical meningioma
108	Tumor cells - cytoplasmic	1	1	Tumor cells - nuclear	2	2	F	44	Atypical meningioma

27	Tumor cells - cytoplasmic	1	1	Tumor cells - nuclear	1	1	M	5	Craniopharyngioma
28	Tumor cells - cytoplasmic	1	1	Tumor cells - nuclear	1	1	M	8	Craniopharyngioma and pilocytoid gliosis
29	Tumor cells - cytoplasmic	1	1	Tumor cells - nuclear	1	1	F	57	Diffuse astrocytoma
30	Tumor cells - cytoplasmic	1	1	Tumor cells - nuclear	1	1	M	83	Diffuse astrocytoma
31	Tumor cells - cytoplasmic	1	1	Tumor cells - nuclear	1	1	M	27	Diffuse astrocytoma
32	Tumor cells - cytoplasmic	1	1	Tumor cells - nuclear	1	1	M	32	Diffuse astrocytoma
33	Tumor cells - cytoplasmic	3	3	Tumor cells - nuclear	1	1	M	48	Diffuse astrocytoma
34	Tumor cells - cytoplasmic	3	3	Tumor cells - nuclear	1	1	F	42	Diffuse astrocytoma
35	Tumor cells - cytoplasmic	3	3	Tumor cells - nuclear	1	1	M	26	Diffuse astrocytoma
36	Tumor cells - cytoplasmic	1	1	Tumor cells - nuclear	1	1	M	19	DNT
37	Tumor cells - cytoplasmic	2	1	Tumor cells - nuclear	1	1	F	35	DNT
38	Tumor cells - cytoplasmic	1	1	Tumor cells - nuclear	1	1	F	58	Ependymoma
39	Tumor cells - cytoplasmic	1	1	Tumor cells - nuclear	1	1	F	24	Ependymoma
40	Tumor cells - cytoplasmic	1	1	Tumor cells - nuclear	1	1	M	18	Ependymoma
41	Tumor cells - cytoplasmic	1	1	Tumor cells - nuclear	1	1	M	63	Ependymoma
42	Tumor cells - cytoplasmic	1	1	Tumor cells - nuclear	1	1	F	13	Ganglioglioma
43	Tumor cells - cytoplasmic	1	1	Tumor cells - nuclear	1	1	F	24	Ganglioglioma
101	Tumor cells - cytoplasmic	2	1	Tumor cells - nuclear	2	1	F	1	Ganglioglioma
44	Tumor cells - cytoplasmic	1	1	Tumor cells - nuclear	1	1	F	11	Glioblastoma
45	Tumor cells - cytoplasmic	1	1	Tumor cells - nuclear	1	1	M	65	Glioblastoma
46	Tumor cells - cytoplasmic	1	1	Tumor cells - nuclear	1	1	F	65	Glioblastoma
47	Tumor cells - cytoplasmic	1	1	Tumor cells - nuclear	1	1	F	57	Glioblastoma
48	Tumor cells - cytoplasmic	1	1	Tumor cells - nuclear	1	1	F	63	Glioblastoma
49	Tumor cells - cytoplasmic	1	1	Tumor cells - nuclear	1	1	M	42	Glioblastoma
50	Tumor cells - cytoplasmic	1	1	Tumor cells - nuclear	1	1	F	3	Glioblastoma
51	Tumor cells - cytoplasmic	1	1	Tumor cells - nuclear	1	1	M	55	Glioblastoma
52	Tumor cells - cytoplasmic	2	1	Tumor cells - nuclear	1	1	F	53	Glioblastoma
53	Tumor cells - cytoplasmic	2	2	Tumor cells - nuclear	1	1	M	54	Glioblastoma
109	Tumor cells - cytoplasmic	1	1	Tumor cells - nuclear	2	2	F	64	Glioblastoma
54	Tumor cells - cytoplasmic	1	1	Tumor cells - nuclear	1	1	F	54	Gliomatosis cerebri
55	Tumor cells - cytoplasmic	1	1	Tumor cells - nuclear	1	1	M	78	Gliosarcoma
56	Tumor cells - cytoplasmic	1	1	Tumor cells - nuclear	1	1	M	59	Gliosarcoma
57	Tumor cells - cytoplasmic	2	1	Tumor cells - nuclear	1	1	M	51	Gliosarcoma
58	Tumor cells - cytoplasmic	1	1	Tumor cells - nuclear	1	1	F	43	Gliosis

59	Tumor cells - cytoplasmic	1	1	Tumor cells - nuclear	1	1	M	9	Gliosis
60	Tumor cells - cytoplasmic	1	1	Tumor cells - nuclear	1	1	M	74	Haemangioblastoma
61	Tumor cells - cytoplasmic	1	1	Tumor cells - nuclear	1	1	M	24	Haemangioblastoma
62	Tumor cells - cytoplasmic	1	1	Tumor cells - nuclear	1	1	M	47	Haemangiopericytoma
63	Tumor cells - cytoplasmic	1	1	Tumor cells - nuclear	1	1	M	2	Medulloblastoma
64	Tumor cells - cytoplasmic	1	1	Tumor cells - nuclear	1	1	M	1	Medulloblastoma
65	Tumor cells - cytoplasmic	1	1	Tumor cells - nuclear	1	1	M	1	Medulloblastoma
66	Tumor cells - cytoplasmic	1	1	Tumor cells - nuclear	1	1	M	14	Medulloblastoma
67	Tumor cells - cytoplasmic	1	1	Tumor cells - nuclear	1	1	M	10	Medulloblastoma
68	Tumor cells - cytoplasmic	1	1	Tumor cells - nuclear	1	1	M	6	Medulloblastoma
69	Tumor cells - cytoplasmic	1	1	Tumor cells - nuclear	1	1	F	63	Meningioma (fibrous)
70	Tumor cells - cytoplasmic	1	1	Tumor cells - nuclear	1	1	F	63	Meningioma (fibrous)
71	Tumor cells - cytoplasmic	1	1	Tumor cells - nuclear	1	1	F	78	Meningioma (meningothelial)
72	Tumor cells - cytoplasmic	1	1	Tumor cells - nuclear	1	1	F	58	Meningioma (meningothelial)
73	Tumor cells - cytoplasmic	1	1	Tumor cells - nuclear	1	1	F	62	Meningioma (transitional)
74	Tumor cells - cytoplasmic	1	1	Tumor cells - nuclear	1	1	F	64	MPNST
102	Tumor cells - cytoplasmic	1	1	Tumor cells - nuclear	2	1	M	28	Myxopapillary ependymoma
103	Tumor cells - cytoplasmic	2	2	Tumor cells - nuclear	2	1	F	28	Myxopapillary ependymoma
75	Tumor cells - cytoplasmic	1	1	Tumor cells - nuclear	1	1	M	0	Neuroblastoma
104	Tumor cells - cytoplasmic	2	2	Tumor cells - nuclear	2	1	F	0	Neuroblastoma
76	Tumor cells - cytoplasmic	1	1	Tumor cells - nuclear	1	1	F	54	Neurofibroma
77	Tumor cells - cytoplasmic	2	2	Tumor cells - nuclear	1	1	F	34	Neurofibroma
78	Tumor cells - cytoplasmic	2	3	Tumor cells - nuclear	1	1	F	53	Neurofibroma
79	Tumor cells - cytoplasmic	1	1	Tumor cells - nuclear	1	1	F	65	Oligodendroglioma
80	Tumor cells - cytoplasmic	1	1	Tumor cells - nuclear	1	1	M	29	Oligodendroglioma
81	Tumor cells - cytoplasmic	1	1	Tumor cells - nuclear	1	1	F	65	Oligodendroglioma
82	Tumor cells - cytoplasmic	1	1	Tumor cells - nuclear	1	1	F	12	Pilocytic astrocytoma
83	Tumor cells - cytoplasmic	1	1	Tumor cells - nuclear	1	1	F	27	Pilocytic astrocytoma
84	Tumor cells - cytoplasmic	1	1	Tumor cells - nuclear	1	1	M	4	Pilocytic astrocytoma
105	Tumor cells - cytoplasmic	2	2	Tumor cells - nuclear	2	1	M	8	Pilocytic astrocytoma
85	Tumor cells - cytoplasmic	1	1	Tumor cells - nuclear	1	1	M	79	Pituitary adenoma
86	Tumor cells - cytoplasmic	2	2	Tumor cells - nuclear	1	1	M	60	Pituitary adenoma
87	Tumor cells - cytoplasmic	2	3	Tumor cells - nuclear	1	1	M	62	Pituitary adenoma
88	Tumor cells - cytoplasmic	2	4	Tumor cells - nuclear	1	1	F	50	Pituitary adenoma

89	Tumor cells - cytoplasmic	3	5	Tumor cells - nuclear	1	1	M	54	Pituitary adenoma
106	Tumor cells - cytoplasmic	2	2	Tumor cells - nuclear	2	1	M	60	Pituitary adenoma
90	Tumor cells - cytoplasmic	1	1	Tumor cells - nuclear	1	1	M	1	PNET
91	Tumor cells - cytoplasmic	2	4	Tumor cells - nuclear	1	1	M	4	PNET
110	Tumor cells - cytoplasmic	2	4	Tumor cells - nuclear	2	4	M	5	PNET
92	Tumor cells - cytoplasmic	1	1	Tumor cells - nuclear	1	1	M	35	Schwannoma
93	Tumor cells - cytoplasmic	1	1	Tumor cells - nuclear	1	1	F	47	Schwannoma
94	Tumor cells - cytoplasmic	1	1	Tumor cells - nuclear	1	1	F	46	Schwannoma
95	Tumor cells - cytoplasmic	1	1	Tumor cells - nuclear	1	1	M	61	Schwannoma
107	Tumor cells - cytoplasmic	2	1	Tumor cells - nuclear	2	1	F	58	Schwannoma
96	Tumor cells - cytoplasmic	1	1	Tumor cells - nuclear	1	1	M	51	Subependymoma
97	Tumor cells - cytoplasmic	1	1	Tumor cells - nuclear	1	1	M	43	Subependymoma
98	Tumor cells - cytoplasmic	1	1	Tumor cells - nuclear	1	1	M	67	Subependymoma

Type: 0 = not set; 1 = negative; 2 = weak; 3 = moderate; 4 = strong. Quantity (cell types): 0 = not set; 1 = 0–1%; 2 = 2–10%; 3 = 11–25%; 4 = 26–50%; 5 = 51–75%; 6 = >75%.

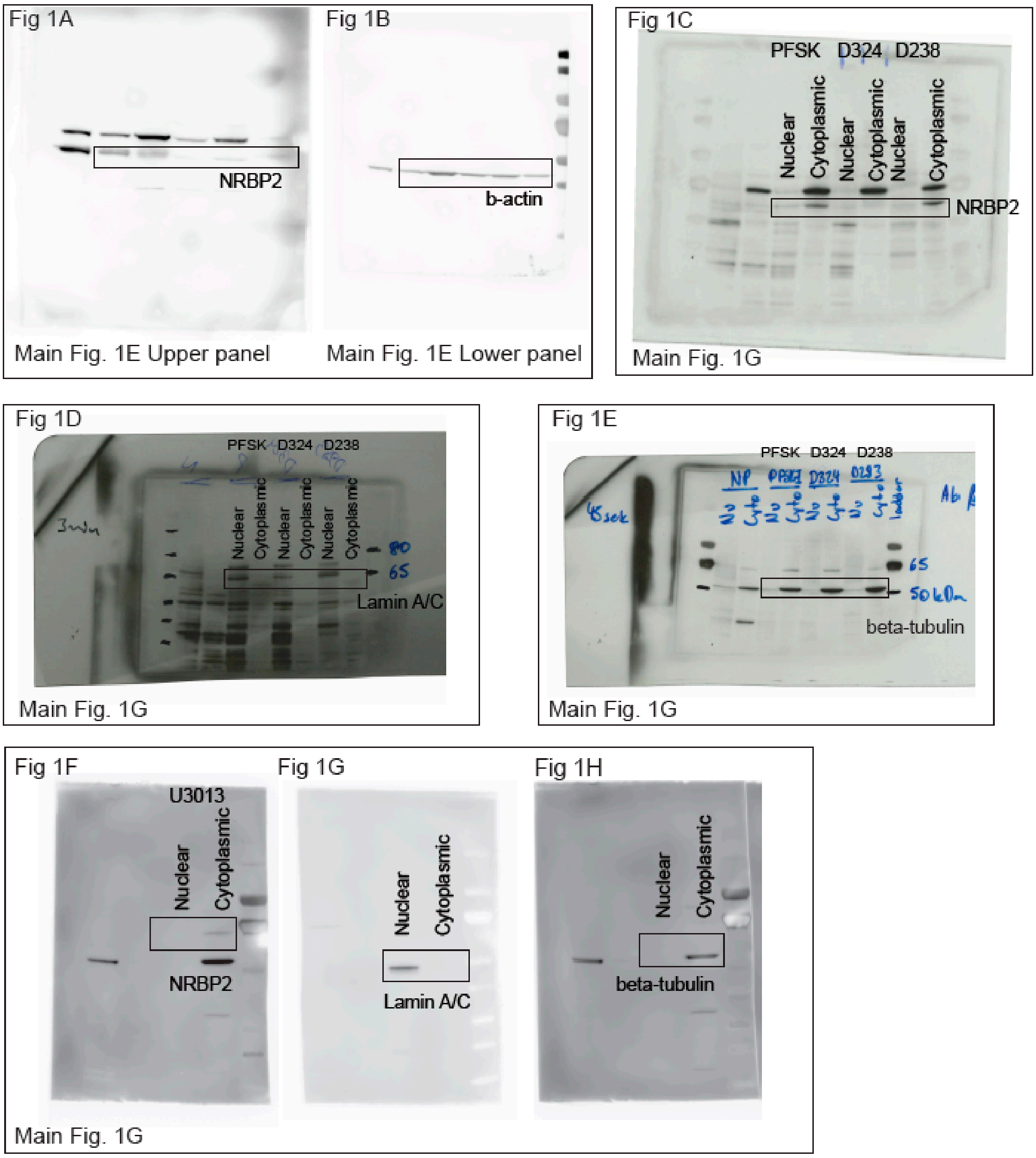

Figure S1. Uncropped western blots for Figure 1.

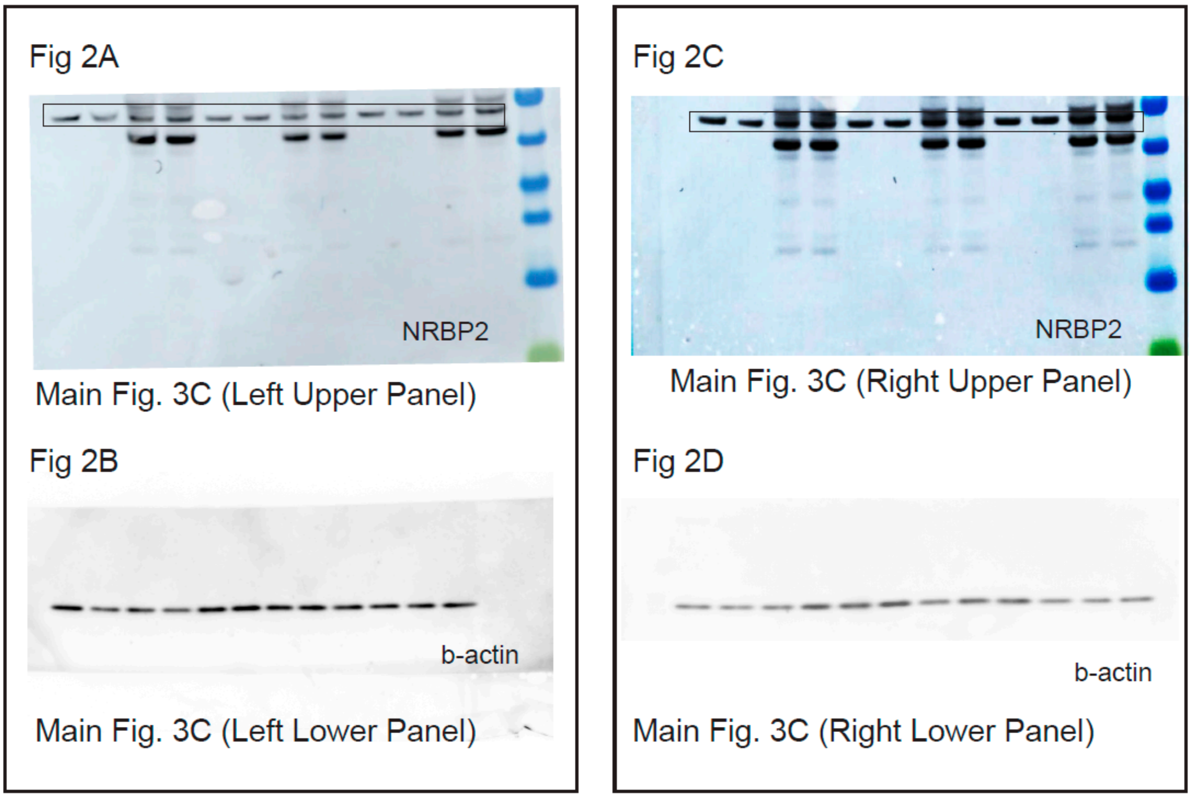

Figure S2. Uncropped western blots for Figure 3.

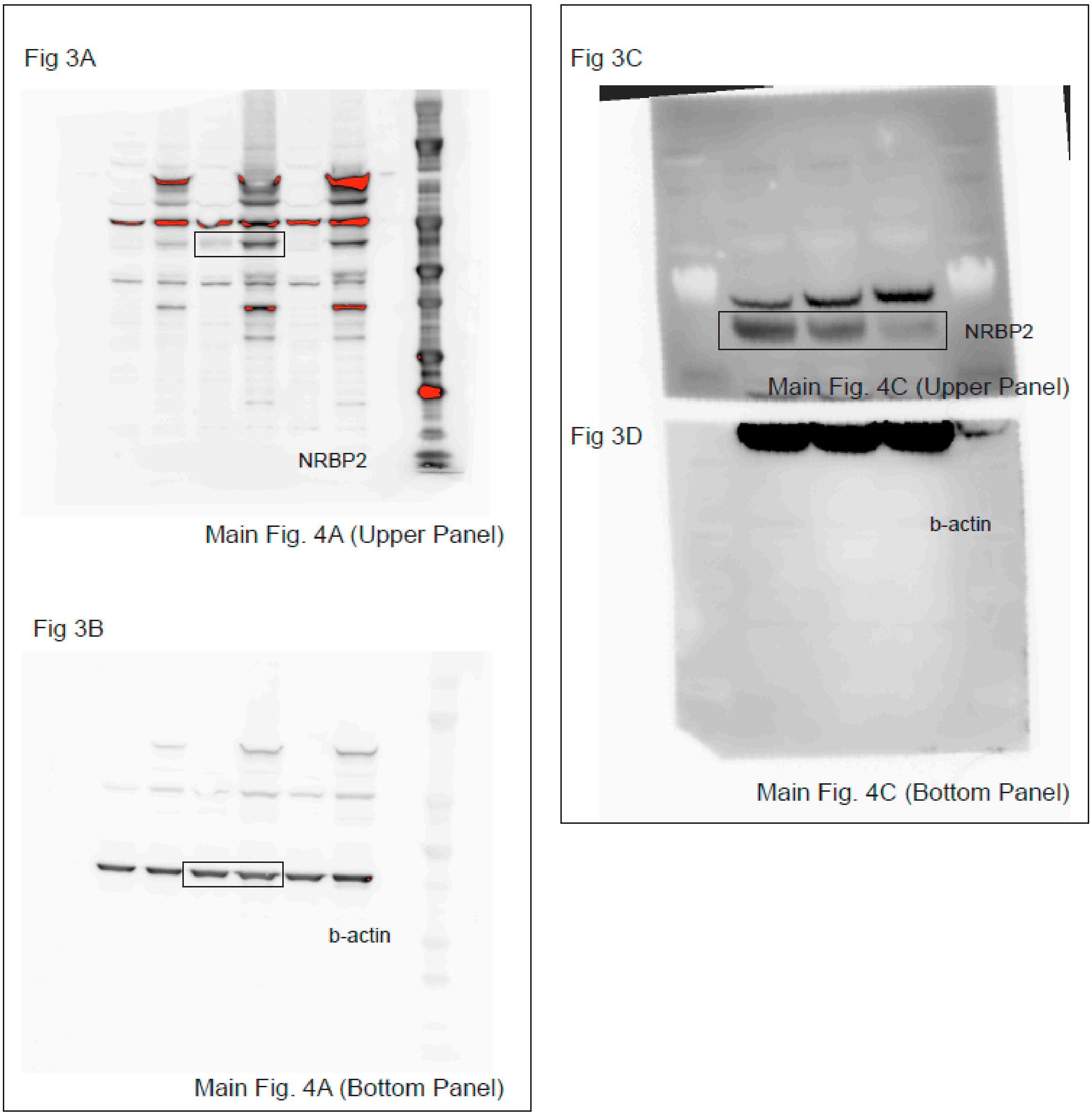

Figure S3. Uncropped western blots for Figure 4.

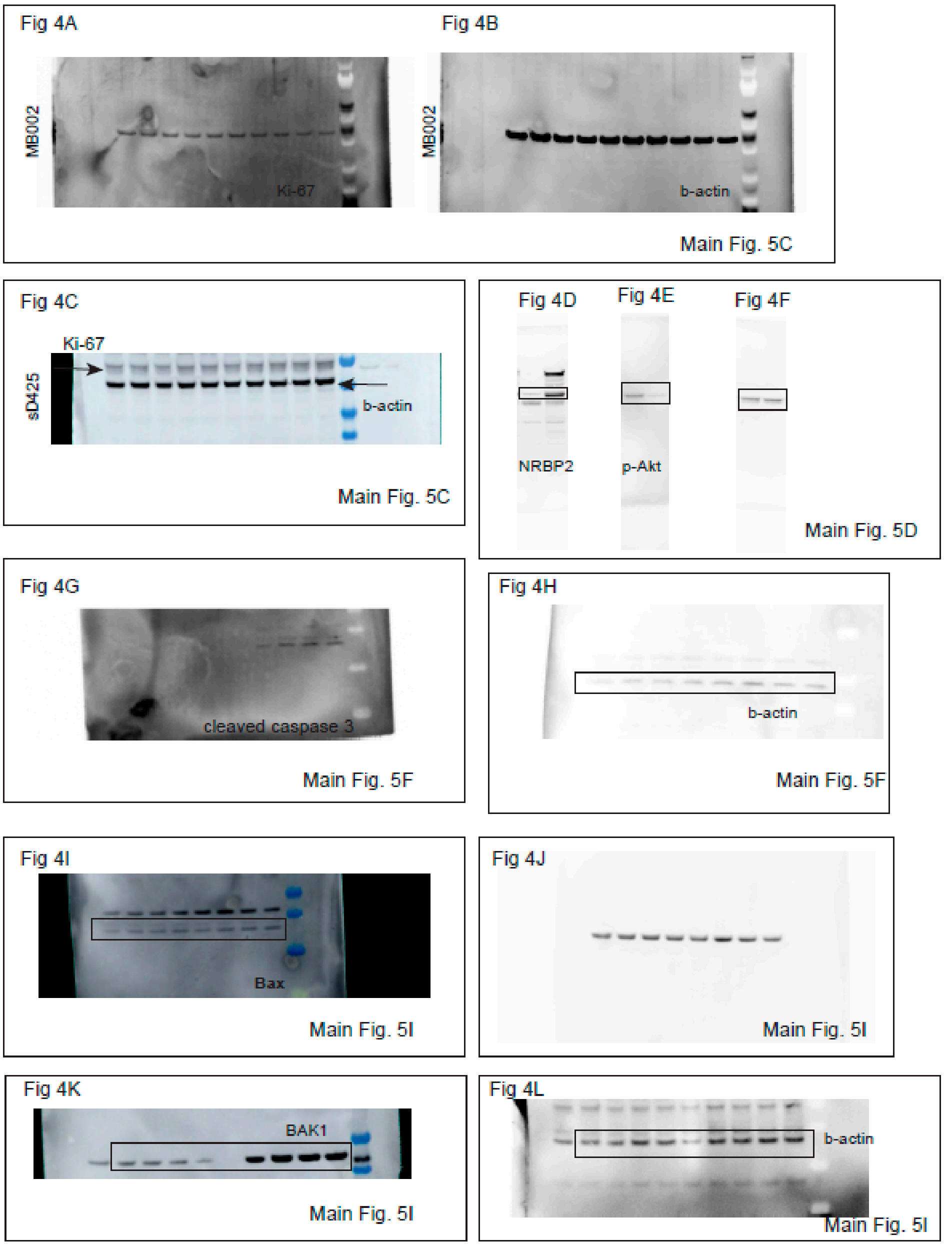

Figure S4. Uncropped western blots for Figure 5.
